# Photocatalytic oxidation of arsenic(iii) in aqueous media: a mini-review

**DOI:** 10.1039/d5ra06908d

**Published:** 2026-01-14

**Authors:** M. Navarrete-Magaña, A. Mantilla, E. Samaniego-Benitez

**Affiliations:** a Instituto Politécnico Nacional, UPIICSA Av. Té 950, Granjas México 08400 Ciudad de México Mexico anavarretem@ipn.mx; b Instituto Politécnico Nacional, CICATA-Legaria Legaria 694, Col. Irrigación 11500 Ciudad de México Mexico; c SECIHTI – Instituto Politécnico Nacional, CICATA-Legaria Legaria 694, Col. Irrigación 11500 Ciudad de México Mexico jose.samaniego@secihti.mx

## Abstract

Prolonged exposure to arsenic (As)-contaminated water poses a serious risk to human health due to its high toxicity, which can cause skin lesions and, in the most severe cases, various types of cancer. It is therefore imperative to develop effective strategies to remove it. However, conventional removal methods have significant limitations for eliminating this metalloid, prompting research into sustainable alternatives, including photocatalytic oxidation. This mini-review examines the most recent advances in photocatalytic oxidation of As(iii) to As(v) species, with a special emphasis on the primary photocatalysts employed, the reaction mechanisms involved, and the operating parameters that determine process efficiency. Various photocatalysts, based on both metal oxides and carbonaceous materials, have shown high efficiencies under ultraviolet and visible irradiation. Likewise, strategies to optimize photocatalytic performance have been explored, such as the construction of heterojunctions and doping with metallic and non-metallic elements, which facilitate charge separation and enhance light absorption, thereby promoting the generation of reactive oxygen species (ROS). Among these, hydroxyl radicals (˙OH) and superoxide radicals (˙O_2_^−^) have been shown to play a key role in the oxidation of As(iii), achieving 100% conversion in a matter of minutes or hours. Finally, recent advances, the advantages and limitations of different photocatalytic approaches, and the main challenges associated with developing robust, economically viable systems for the treatment of arsenic-contaminated water are analyzed.

## Abstract 2

This review summarizes recent advances in photocatalytic As(iii) oxidation, highlighting semiconductor engineering, metal oxide and carbon-based materials, and emerging strategies to improve light utilization and charge separation.

## Graphical abstract

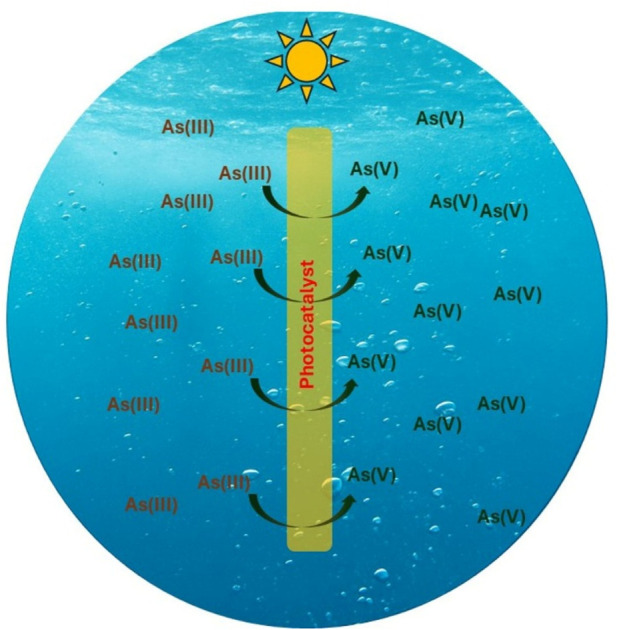

## 1. Introduction

Arsenic is one of the most toxic and dangerous elements present in the environment, representing a significant threat to public health globally.^1^ It is estimated that its exposure affects more than 200 million people worldwide.^2^ The sources of arsenic are both anthropogenic and natural. Anthropogenic sources include the intensive use of pesticides in agriculture, especially in cultivating fruit and vegetables, which can lead to food contamination for human consumption.^3,4^ In addition, arsenic also originates naturally from geological processes, such as volcanic activity, which releases arsenic-rich particles into the atmosphere.^5–7^ These particles are subsequently deposited in the soil and can infiltrate subway aquifers, a primary water source used for both food preparation and personal consumption.^8^ Arsenic in its trivalent oxidation state (As(iii)) is considerably more toxic and has higher mobility in aquatic systems compared to its pentavalent form (As(v)), making it difficult to remove by conventional water treatment technologies.^9,10^ Although effective under certain conditions, techniques such as adsorption, coagulation-filtration, and reverse osmosis show significant limitations in As(iii) removal,^11^ mainly due to their low chemical reactivity.^12^ In this context, heterogeneous photocatalysis has emerged as a promising alternative for the oxidation of As(iii) to As(v), thus facilitating its subsequent separation from the aqueous medium. This process is based on activating semiconductor materials by luminous radiation (visible or ultraviolet), generating reactive oxygen species (ROS) that promote the efficient conversion of As(iii).^13^ In addition, photocatalysis offers significant operational advantages, such as low operating costs, room temperature operating conditions, and high scalability potential for large-scale applications. Various semiconductor materials have been investigated in the field of photocatalytic oxidation of As(iii), including oxides such as WO_3_, TiO_2_, and Fe_2_O_3_, among others.^14,15^ Given the increasing volume of published research in this field, it is timely to perform a mini-review synthesizing the most relevant advances, with emphasis on the oxidation mechanisms, the photocatalysts used, and the experimental conditions that optimize the conversion of As(iii) to As(v). In this context, the present article provides a brief and updated review of the photocatalytic oxidation of As(iii) in aqueous media, highlighting the main technical challenges and prospects for future development in this area of research.

## 2. Exposure effects on arsenic(iii)

**Fig. 1:**
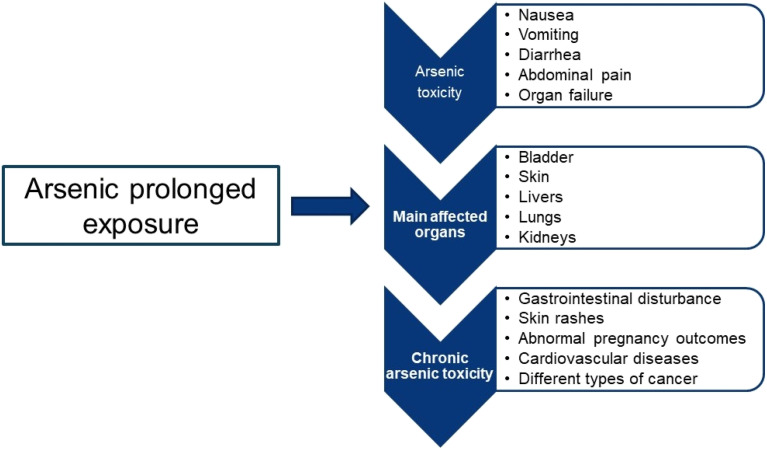
Chronic toxic effects associated with prolonged exposure to arsenic.

As is a metalloid with 33 atomic numbers, which possess properties both metallic and nonmetallic. In the environment, it can be found commonly in inorganic form, mostly in oxyanion form. It can exist in various oxidation states, the most relevant being As(iii) and As(v). The concentration and form of As in aquifers depend principally on: (I) redox conditions (oxidizing or reducing), (II) pH of water (adsorption and mobility of the As species), (III) mineralogical composition of aquifers, (IV) human activity (mining, pesticides, industrial water residues). Due to its high toxicity, especially in the As(iii) species form, presence of As in water potable represents a serious risk to public health. For this reason, organisms like OMS have established a maximum limit of 10 µg L^−1^ for drinking water. Nevertheless, this margin is exceeded greatly in different countries worldwide.^16^ Exposure of As(iii) is a problem of public global health due to its presence in water, soil and foods, and its capacity to cause toxicity in multiple systems. As prolonged exposure is linked to skin diseases such as hyperkeratosis, abnormal pigmentation, and can mainly affect organs such as liver, kidneys, skin and lungs. Non-communicable diseases are also associated, such as type 2 diabetes mellitus (due to β-cell dysfunction and insulin resistance) and cardiovascular diseases (hypertension, arteriosclerosis, heart attack, and vascular dysfunction). Neurological and cognitive effects, immune disorders, and increased infant mortality and morbidity in child development are also reported.^17,18^ Recent studies have advanced our understanding of the cellular and molecular mechanisms responsible for these toxic effects Recent investigation has deepened in the cellular and molecular mechanisms that underlying to these toxic effects.^19^ As reported by Shiblur, prolonged exposure to arsenic is associated with the development of chronic toxicity, which manifests itself in gastrointestinal alterations, complications during pregnancy, and, in more severe scenarios, with the appearance of various types of malignant neoplasms.^20^Fig. 1 shows a diagram with the summary of these effects.

Olubusayo *et al.*, on their part, found that arsenic disrupts cellular metabolism by inhibiting key enzymes, such as pyruvate dehydrogenase (PDH), thereby uncoupling oxidative phosphorylation and reducing ATP production, which leads to lactic acidosis and cellular distress. In addition, it interferes with insulin signaling by blocking the PDK1/Akt pathway and decreasing the expression of Sirt3, which affects PGC-1α and HO-1, thereby increasing oxidative stress and mitochondrial dysfunction. In terms of genotoxicity, arsenic generates reactive oxygen species (ROS) that cause DNA damage, such as chain breaks, micronuclei, chromosomal aberrations, and DNA hyper- or hypomethylation, in addition to inhibiting repair proteins like ERCC1 and PARP1, which promotes carcinogenesis.^21^ Qianying *et al.* studied exposure to arsenic at environmentally relevant levels (0.25 and 1.0 ppm) during postnatal development and found that it induces significant metabolic toxicity in a mouse model. Our findings, supported by integrated transcriptome and metabolome analyses, show pathological alterations in the liver and intestine, disruption of the intestinal barrier, oxidative stress, and hepatic lipid accumulation. At the transcriptomic level, dysregulation of key genes in mitochondrial β-oxidation (Cpt1a, Cpt2, Hadha, Acadl, Acox1) was identified, while the metabolomic profile revealed biomarkers such as l-palmitoylcarnitine. These changes reflect a molecular signature like that observed in patients with non-alcoholic fatty liver disease (NAFLD), reinforcing the translational relevance of the model.^22^ Sen Wei *et al.* chronic exposure to arsenic is a significant risk factor for pancreatic dysfunction and type 2 diabetes. In murine models and MIN6 cells, arsenic was shown to induce ferroptosis, a form of iron-dependent cell death and lipid accumulation, through the generation of mitochondrial ROS (MtROS) and mitochondrial dysfunction (degradation of membrane potential and decrease in cytochrome c). The elimination of MtROS with Mito TEMPO and the inhibition of autophagy or ferritinophagy reduced ferroptosis. These results indicate that arsenic activates pancreatic ferroptosis through a mitochondrial ROS-autophagy–lysosomal pathway, which compromises iron homeostasis and contributes to pancreatic dysfunction.^23^

## 3. Technologies for arsenic oxidation

There is a wide range of research and technological development focused on the treatment of arsenic-contaminated water. These research and developments are aimed at reducing the toxicity and mobility of contaminants before disposal. Traditional strategies for As(iii) removal have been based on physical, chemical, or combined processes, which differ in their operating principles, costs, and efficiency. Physical methods include membrane filtration, adsorption, and reverse osmosis, all of which operate by separating or retaining the contaminant without altering its oxidation state. For these technologies, typical removal efficiencies exceed 90%. For example, a coagulation-filtration process using ferric chloride can remove more than 90% of As(v) from water,^24^ while an anion exchange resin can remove 80% of As(vi) from water.^25^ On the other hand, aluminum-loaded zeolite adsorption media can remove 90% of As(v),^26^ and reverse osmosis (RO) can remove more than 95% of As(v) from water.^26^ However, all the technologies mentioned exhibit a drastic decrease in As(iii) removal efficiency because most As(iii) is present as non-ionized, neutral arsenite acid at typical groundwater pH levels. As(iii) removal typically achieves between 30% and 60% through adsorption, ion exchange, and reverse osmosis.

**Fig. 2:**
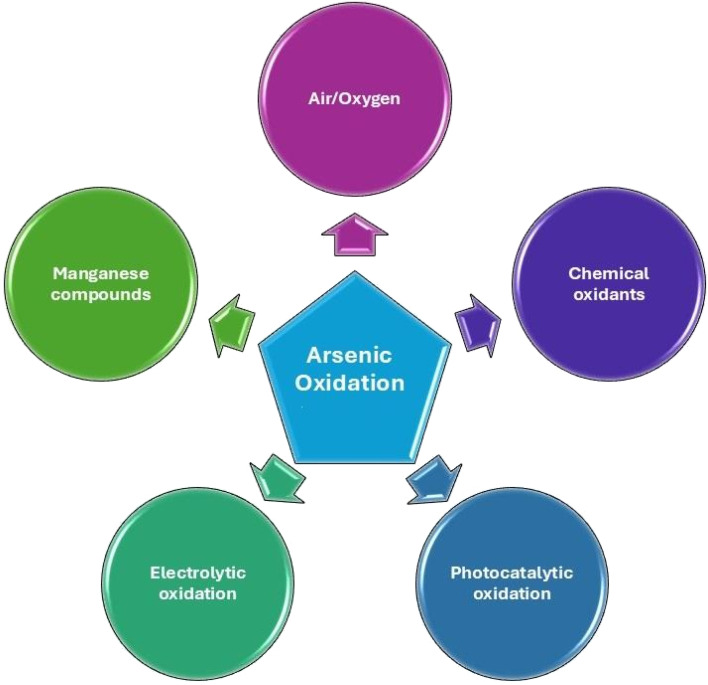
Common processes reported for the oxidation of As(iii) to As(v) in water.

To improve arsenite removal, As(iii) can be oxidized to As(v), and then treated using ion exchange, adsorption, coagulation, or reverse.^27^ The most reported As(iii) oxidation methods are: oxidation in air or oxygen, with the use of chemical oxidants, using manganese compounds, by electrocatalysis and by photocatalysis (Fig. 2).

Although the oxidation of As with air or oxygen has advantages such as easy storage and ease of the process, the process is very slow, taking even days to reach a considerable percentage of oxidized arsenic.^28^ In experiments conducted in the presence of air, a conversion of 25% of As(iii) was achieved after five days of reaction, while using pure oxygen resulted in 8% oxidation in one hour.^29^

To increase the oxidation kinetics of As(iii), oxidizing agents such as ozone, chlorine, hydrogen peroxide, *etc.* are used. The use of ozone has shown efficiency in the As(iii) oxidation in a wide range of pH values as reported by Khuntia *et al.*^30^ In their work, the authors managed to generate ozone microbubbles in solution, which gave them an efficiency of 95% at different As(iii) concentrations between 50 and 200 µg L^−1^. In the work done by Amiri *et al.*, sodium hypochlorite (NaClO), hydrogen peroxide and ozone were used as oxidizing agents for As(iii) oxidation in aqueous solution.^31^ Their study showed that, although the 3 oxidizing agents manage to oxidize As(iii), NaClO is the best, achieving 99% efficiency in less than 5 minutes. However, although the use of oxidizing agents improves the kinetics of As(iii) to As(v), it is necessary to consider the substances present in the water when selecting the oxidizing agent, since these substances can affect the kinetics of the oxidation of As(iii).^32^ It has been seen that the efficiency of As(iii) oxidation using ozone is considerably reduced if carbon compounds or S^2−^ ions are present in the water.^33^

Among various nanomaterials, manganese oxides (MnOx) have emerged as the most promising for water purification due not only to their stability, low cost, efficiency and ease of synthesis in an environmentally friendly manner,^34,35^ but also Mn-containing oxides are strong oxidants that participate in extensive redox reactions with inorganic/organic chemical species. Similarly, MnOx also has high adsorption capacities for different ions with the ability to disperse and change the bioavailability of various poisonous and important elements.^36^ Therefore, MnOx is widely used as an oxidant for the oxidation of As.

**Fig. 3:**
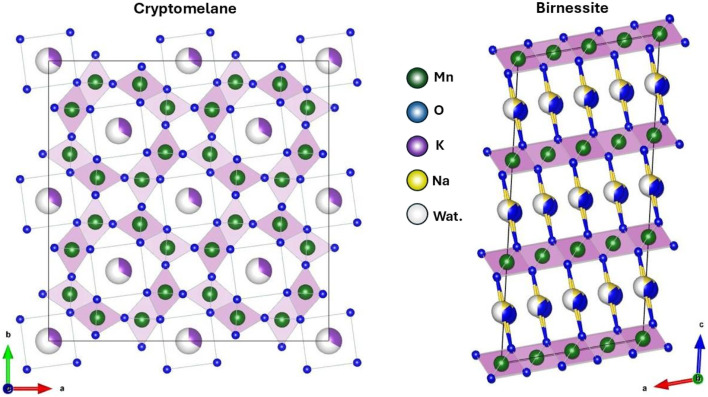
Crystal structures of manganese oxides. (left) Tunnel-like structure of cryptomelane, showing channels that can accommodate cations such as K^+^ or Na^+^. (right) Layered structure of birnessite with ions and water molecules in the interlayer space. These structures directly influence the adsorption capacity and photocatalytic activity for the oxidation of As(iii). Tunnel structure of cryptomelane and laminar structure of birnessite.

Among the different structures that MnOx has, cryptomelane and birnessite are the most reported for the removal and oxidation of As(iii) (Fig. 3). Cryptomelane is a mixed oxide of potassium and manganese consisting of a tunneled structure made up of double chains of MnO_6_ octahedra that share vertices. The tunnel size is approximately 0.46 × 0.46 nm, and K ionic species and water molecules are housed inside, providing structural stability.^37^ One of the advantages of this phase of MnOx is that the structure and composition can be easily determined by inexpensive laboratory procedures leading to a synthetic material commonly referred to as 2 × 2 octahedral molecular sieve (OMS-2).^38–40^ Meanwhile, birnessite is composed of Mn–O octahedra that form octahedral layers like clays, and has Na^+^, Ca^2+^ or K^+^ ions in the region called interlayer surrounded by water molecules that compensate the electrical charge of its layers.

Hou and collaborators succeeded in synthesizing OMS-2 nanostructures with different amounts of oxygen vacancies through the hydrothermal process.^41^ The results of their experiments showed that increasing the concentration of oxygen voids in OMS-2 not only greatly increases the oxidation activity of As(iii) in aqueous medium (reaching a kinetics of 14.6 µmol g^−1^ min^−1^), but also prevents the unfavorable effect of coexisting ions of As(v), phosphate, Mn^2+^ and Fe^2+^ species, *etc.* Similarly, two years later, Hou *et al.* demonstrated that the high concentration of K^+^ in the OMS-2 structure significantly enhances the oxidative activity of As(iii) and increases the reaction rate, thus reducing the adverse effect of coexisting ions such as As(v) and phosphate.^42^ Some authors have mixed OMS-2 with other materials to increase the removal of As(iii) in water, such as Jakkapop Phanthasri and collaborators who observed an increase in the removal of As(iii) by combining OMS-2 with iron-benzenetricarboxylate.^43^ Generally, the oxidation reaction of As(iii) to As(v) can take from minutes to hours using birnessite.^44,45^ Several authors have focused their studies on the effect on the oxidative properties of bernisite in the presence of different low molecular weight organic acids produced by plants, bacteria and fungi, which are always present in the aquatic environment, in order to simulate an application in a real environment. Mengyu Liang and co-workers studied the effect of the presence of citrate and EDTA during the oxidation reaction of As(iii), finding that citrate inhibited As(iii) oxidation and As(v) adsorption, while EDTA promoted As(iii) oxidation.^46^

**Fig. 4:**
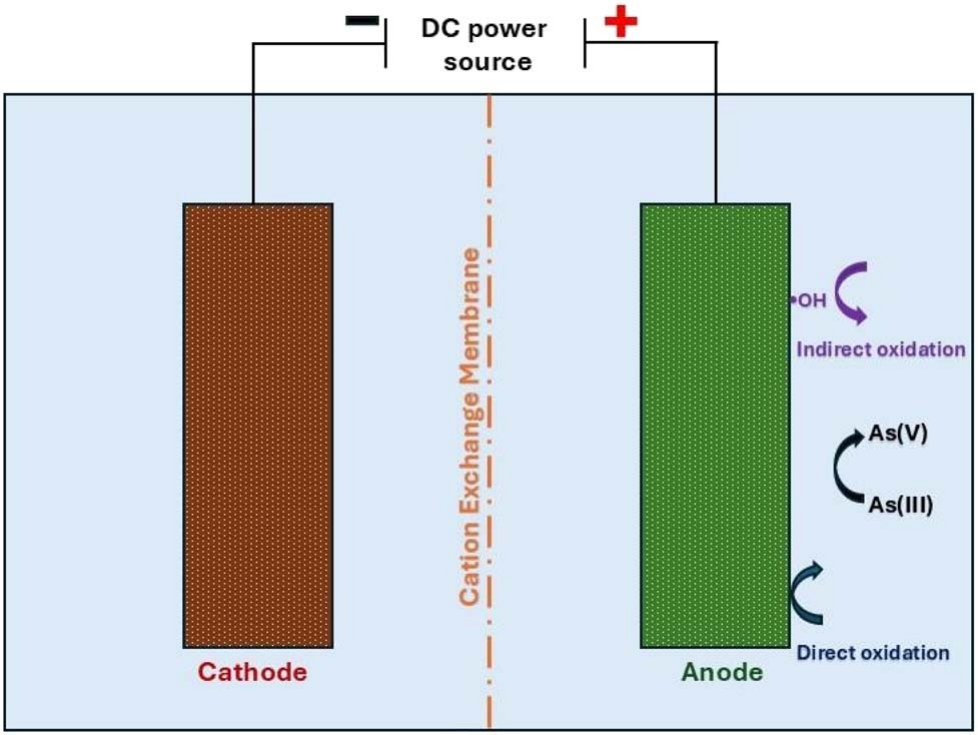
Representative scheme of arsenic oxidation by the electrochemical process.

Electrochemical oxidation (EO), see Fig. 4, has been gaining considerable interest in recent years and has shown potential to replace the use of chemical oxidants such as KMnO_4_, HOCl, H_2_O_2_/UV and O_3_, for the removal of organic/inorganic compounds for disinfection purposes.^47–49^ Among the electrochemical oxidation techniques, anodic oxidation (AO) is considered possibly the most popular and applicable from a practical perspective.^50^ In the anodic oxidation process, contaminants can be oxidized by two main routes: (a) direct surface oxidation by electron transfer and (b) indirect oxidation by generated oxidizing agents. Indirect oxidation can be either by the formation of OH radicals found in the vicinity of the electrode surface, or by indirect oxidation by oxidizing agents generated from ions available in the bulk solution (for example, chlorine from chloride).^49,51^

In the anionic oxidation process, the material used as anode is one of the most important parameters. Depending on the selectivity and oxygen evolution capacity, anode materials are classified into (a) active anode (with the ability to produce relatively low amounts of over potential oxygen evolution that favor the partial and selective oxidation of contaminants) and (b) non-active anode (produce large amounts of over potential oxygen and have the ability to complete oxidation in a non-specific manner).^52^ Platinum is considered one of the best anode metals with an oxidation rate of arsenic of 90–100%, which is excellent, rapid and highly selective for catalyzing the conversion of As(iii) to As(v).^53,54^ Likewise, the combination of metals such as platinum (Pt) and gold (Au) has proven to be excellent anodes for the oxidation of As. Diep Vu Ca and his collaborators deposited Pt and Au nanoparticles on indium tin oxide (ITO) electrodes. The modified ITO was used in the oxidation of As(iii), achieving nearly complete efficiency in the concentration range of 0.2–1.0 mmol L^−1^. The authors noted that the efficiency of the oxidative process depended on the nanoparticles' geometry and nonuniform size, as well as on the electron-transfer sites formed during electrode modification.^55^ Despite their high efficiency, the use of platinum and other noble metals is not practical for large-scale use due to their high cost, so many researchers have been testing different materials as electrodes. Among the different materials studied, titanium dioxide (TiO_2_) is a promising material for the removal of As from water due to its physical and chemical stability, low toxicity, corrosion resistance and the fact that it contains a strong oxidizing power of its holes.^56^ TiO_2_ is generally mixed with other metal oxides or metals to increase the oxidative capacity. For example, Y. Xiong and collaborators managed to synthesize a titanium-based anode coated with MnO_2_/TiO_2_, nanotubes which showed a high oxidation efficiency of As(iii) (approximately 90%) at an As(iii) concentration of 1 mg L^−1^ after 120 minutes of electrolysis.^57^ Peng Zhang and co-workers successfully synthesized a Ti/TiO_2_NTs/Sb–SnO_2_, composite anode by combining anodization and sol–gel methods, the electrode showed a consistently high electrochemical activity for As(iii) oxidation: 6.67 µM As(iii) was oxidized to As(v) in 60 min by direct electron transfer.^58^

**Fig. 5:**
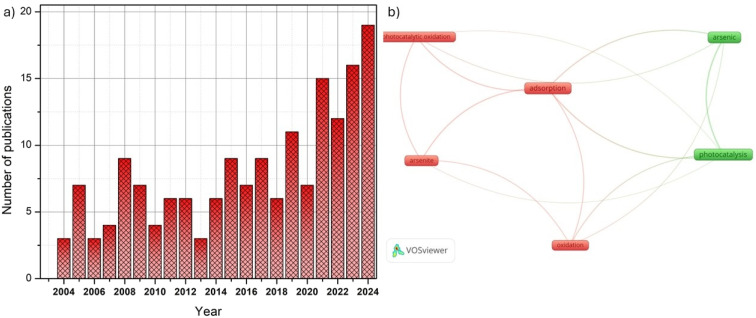
(a) Number of publications in the last 20 years obtained from Scopus using the combined keywords “arsenic oxidation” + “photocatalysis”. (b) Keyword co-occurrence network generated with VOSviewer.

**Fig. 6:**
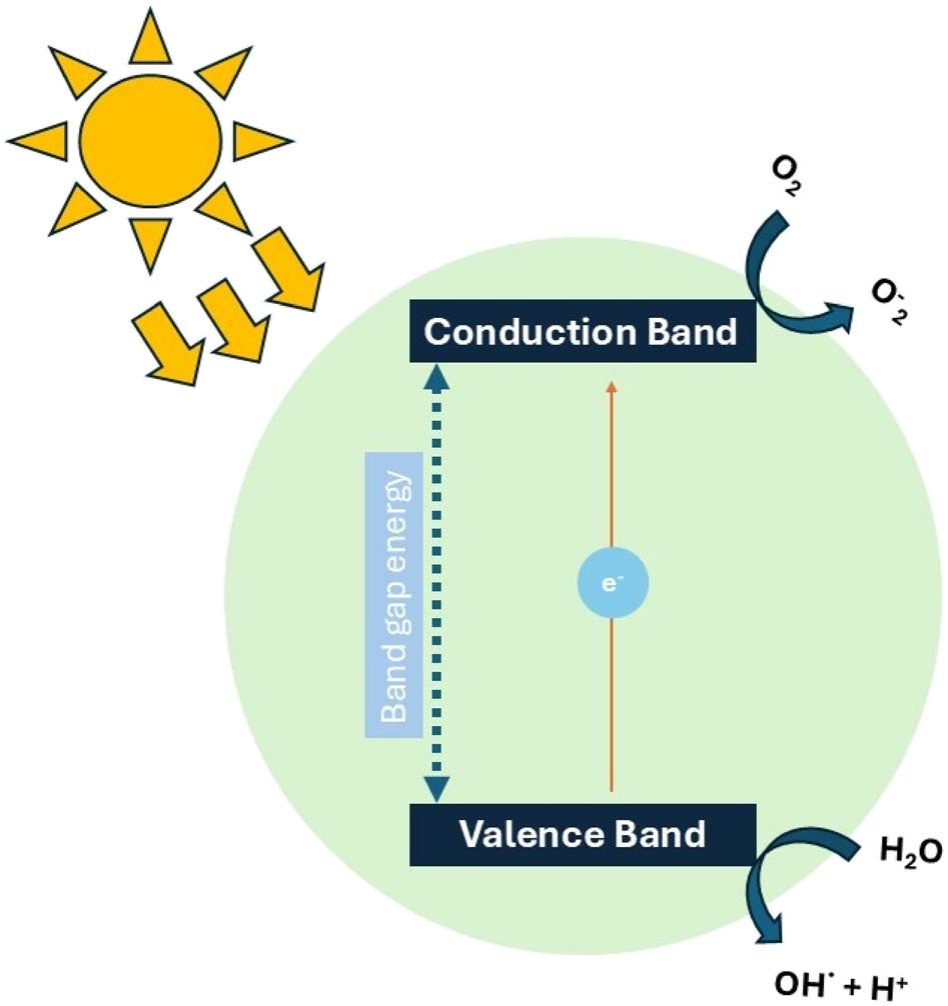
Schematic mechanism of the photocatalysis process. Under light irradiation, an electron (e^−^) from the photocatalyst is excited from the Valence Band (VB) to the Conduction Band (CB), generating a hole (h^+^) in the VB. This hole can react directly with water molecules to generate hydroxyl radicals (OH^+^). Simultaneously, e^−^ in the conduction band reduces O_2_ to produce superoxide radicals (˙O_2_^−^): both species contribute to the oxidation of water molecules.

One of the most recently investigated methods for As oxidation is heterogeneous photocatalysis. Although this technique has been widely applied to the oxidation or degradation of organic pollutants,^59^ its application to arsenic oxidation has been relatively limited. In the last 20 years, approximately 170 articles have been published using the combined keywords “arsenic oxidation + photocatalysis” (Fig. 5a). Despite this relatively small number, a clear upward trend has been observed in recent years, indicating growing scientific interest in this topic. To identify the main lines of research and their interrelationships with respect to existing research on this topic, a keyword co-occurrence map was created using VOSviewer (Fig. 5b). The analysis revealed two predominant clusters: one linked to photocatalysis and the other to adsorption. This distribution reflects the recent trend toward combining oxidation and adsorption processes as complementary strategies for arsenic removal. One of the most recent methods studied for the oxidation of arsenic is the process of heterogeneous photocatalysis. In general, semiconductor materials such as metal oxides or sulfides, carbon-based materials, among others, are used in the photocatalysis process. When the semiconductor material is irradiated with an energy greater than or equal to the band gap energy between the semiconductor valence band (VB) and the conduction band (CB), the electrons (e^−^) located in the VB can jump to CB and can leave a positively charged hole (h^+^) in the VB. This pair of h^+^ and e^−^ can, respectively, migrate to the surface of the semiconductor to undergo a series of oxidation and reduction reactions, which materialize in the conversion of different valence states in the treatment of heavy metals (Fig. 6).

The aim of this review is to summarize the progress made in the oxidation of As(iii) through the photocatalytic process. The design of smart materials to solve some general problems in photocatalysis, and particularly in the oxidation of As(iii), is discussed in detail.

## 4. Photocatalytic performance in arsenic(iii) oxidation

### 4.1. Ti-based photocatalysts

According to previous studies, several examples of the oxidative removal of As(iii) through heterogeneous photocatalysis processes using TiO_2_ as a photocatalyst have been reported, activated under both ultraviolet and visible radiation. It has been observed that the oxidation of As(iii) occurs efficiently under neutral pH conditions (6–8). The reported reaction times range from 10 to 60 minutes for initial arsenic concentrations of 5 to 40 ppm. In addition, the efficiency of the process can be significantly increased when TiO_2_ is modified by doping with metals or by incorporating mixed oxides, which favors charge separation and the generation of reactive species.

Hany *et al.* reported reduced quantum yield in the photocatalysis of As(iii) using TiO_2_, expressed as a low number of molecules oxidized per absorbed photon, which demonstrates the limited intrinsic efficiency of the system. This low efficiency suggests that the pure photocatalyst has limitations in generating and effectively utilizing reactive species. Consequently, structural modification of TiO_2_ is proposed, either by doping with metallic or non-metallic elements, or by forming heterostructures.^60^ In a study conducted by our research group, doping with WO_3_ particles using the sol–gel method enabled the complete oxidation of As(iii) to As(v) starting from an initial concentration of 10 ppm in 25 minutes under UV irradiation. It was determined that the hetero-structural coupling between TiO_2_ and WO_3_ significantly improved the efficiency of photoinduced charge separation and transfer at the interface between the two oxides. This improvement was attributed to the reduction in crystallite size, the increase in specific surface area, and the broadening of the spectral absorption range, extending from the ultraviolet to the visible region. These factors synergistically contributed to the increased photocatalytic efficiency of the hybrid system.^61^ Xiaoxiao *et al.* investigated the influence of the size and electronic configuration of TiO_2_-supported nanocatalysts on the efficiency of photocatalytic oxidation of As(iii). The results indicated that reducing the particle size modifies the distribution of orbitals in surface atoms, which favors the adsorption and activation of reactive species such as As(iii) and H_2_O_2_. In addition, it was demonstrated that the introduction of oxygen vacancies and doping with different elements improves the separation of photoinduced charges, increasing the generation of reactive oxygen species (ROS), which are responsible for oxidation. The study also revealed that specific structural configurations, including oxide heterostructures and size-optimized nanocatalysts, favor catalytic pathways characterized by hydroxyl radical (OH) dominance. These highly reactive species play a central role in the efficient conversion of As(iii) to As(v).^62^ Adreina *et al.* developed a photocatalytic system based on the impregnation of anatase-phase TiO_2_ (7%) onto a preformed mesoporous SBA-15 matrix, obtaining the Ti-SBA-15 material. Under irradiation conditions, the system demonstrated a conversion rate of more than 98% from As(iii) to As(v), regardless of the solution's pH. In addition, significant adsorption capacity was observed, reaching up to 30% in alkaline media. The study identified the formation of reactive oxygen species, mainly hydroxyl radicals (˙OH) and singlet oxygen (^1^O_2_), as the dominant oxidizing agents in the conversion process. These results demonstrate the effectiveness of the Ti-SBA-15 material as a photocatalyst for arsenic remediation in aqueous systems.^63^ Maibelin Rosales *et al.* evaluated the performance of TiO_2_ as a bifunctional material in the removal of As(iii) through simultaneous photoinduced oxidation and adsorption processes. The study revealed that both TiO_2_ nanoparticles (TNP) and nanotubes (TNT) in the anatase phase possess a dual capacity to oxidize As(iii) to As(v) and efficiently adsorb the oxidized product, particularly under alkaline conditions, which are favored by a high density of surface hydroxyl groups. However, nanotubes exhibited greater photoreactivity compared to nanoparticles, attributed to their one-dimensional morphology, which improves the separation of photoinduced charges (electron–hole pairs) and promotes the formation of hydroxyl radicals (OH). These findings highlight the influence of the nanometric morphology of TiO_2_ on the efficiency of remediation mechanisms.^64^ As can be seen, the photocatalytic conversion of trivalent arsenic species to their pentavalent form using TiO_2_ as a catalyst represents a significant challenge in the field of environmental remediation. One of the main limiting factors of the process is the rapid recombination of electron–hole pairs generated during irradiation, which significantly reduces oxidation efficiency. In addition, TiO_2_ can only be activated by radiation in the ultraviolet range, which restricts its use of available sunlight. In addition, in its pure form, TiO_2_ shows a limited capacity to oxidize As(iii), commonly requiring structural or compositional modifications. Among these strategies, doping with metals or non-metals, as well as the formation of heterostructures, stand out, which aim to enhance charge transfer between charge carriers. Several studies have confirmed that the oxidation of As(iii) does not occur directly, but instead progresses through a series of steps involving individual electronic transfers or radical-like species. Such processes give rise to short-lived intermediates, which eventually transform into As(v).

**Fig. 7:**
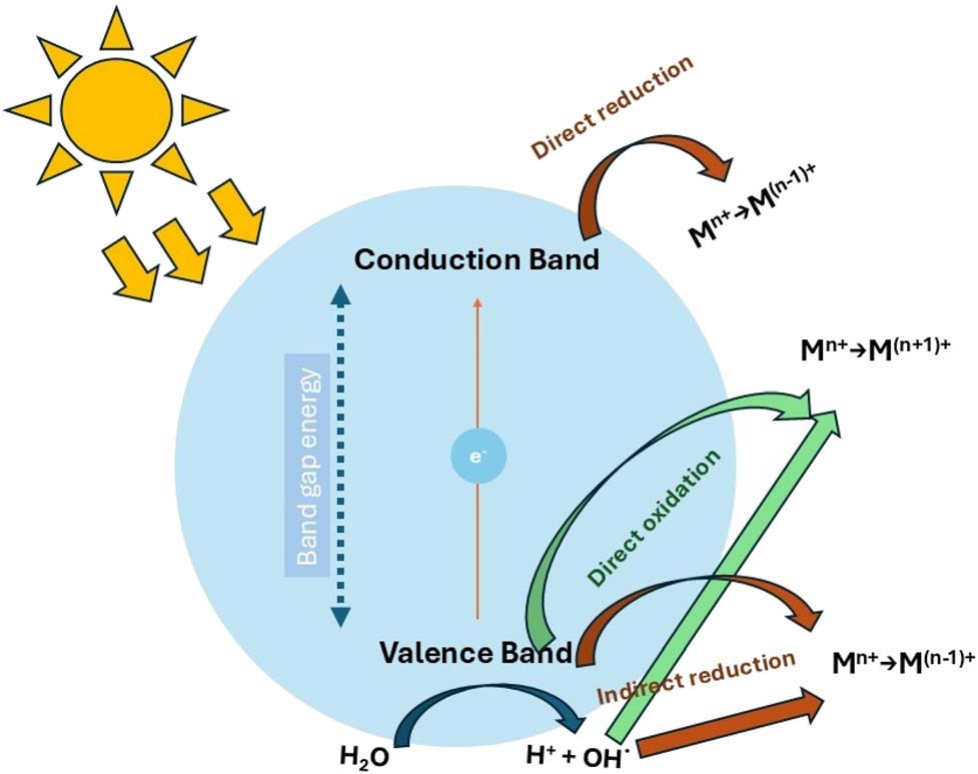
Conceptual representation of the process by which metal ions are transformed photocatalytically in the presence of TiO_2_: direct and indirect oxidation pathways.

According to the review by Litter *et al.*,^65^ this conversion can be explained by a multistage mechanism depicted in Fig. 7, adapted from their work. In particular, Fig. 7 shows the main events involved in photocatalysis using TiO_2_ for the photocatalytic removal of metal ion (M^*n*+^) species. The scheme poses three possible routes: (1) a reduction facilitated by the direct action of photogenerated electrons; (2) an indirect oxidation, mediated by holes or hydroxyl radicals capable of transforming electron donors present in the solution; and (3) a direct oxidation promoted by holes or hydroxyl radicals.

### 4.2. Zn based photocatalysts

Zinc oxide (ZnO) is a white n-type semiconductor widely used in photocatalytic processes for the degradation of organic contaminants such as dyes and phenolic compounds. ZnO can occur in three crystalline phases: hexagonal wurtzite, cubic zinc blende, and cubic rock salt.^66^ The wurtzite structure is the most common and stable under ambient conditions due to its ionicity, which lies exactly at the boundary between covalent and ionic materials. ZnO has a band gap of 3.37 eV (similar to TiO_2_) with conduction and valence band positions of −0.5 V *vs.* NHE and 2.7 V *vs.* NHE, respectively. These positions allow the O_2_/˙O_2_^−^ (−0.33 V *vs.* NHE) and ˙OH/H_2_O (2.53 V *vs.* NHE) reactions, which generate a large number of oxidizing species in water.

Research has shown that the oxidation of As(iii) by heterogeneous photocatalysis using ZnO is possible. Rivera-Reyna and collaborators managed to oxidize a concentration of 5 ppm of As(iii) in 120 minutes under UV irradiation (350 nm) using ZnO synthesized by the sol–gel route.^67^ The authors compared the As(iii) photooxidation performance of synthesized ZnO against commercial ZnO and Degussa TiO_2_; the results showed that the synthesized ZnO had a better photocatalytic performance than commercial ZnO but slightly lower than TiO_2_, however the synthesized ZnO showed a high adsorption of As(vi), removing it completely from the water. On the other hand, Adnan and coworkers created a ZnO coating on ceramic plates using the dip-coating technique,^68^ achieving a homogeneous coating. The coatings exhibited efficient oxidation of As(iii) after 60 minutes of irradiation at 265 nm (Table 1).

**Table 1:** Comparison of ZnO-based photocatalysts for As(iii) oxidation: synthesis methods, experimental conditions and efficiency

Catalyst	Synthesis method	Experimental conditions	Irradiation	As(iii) oxidation	Ref.
ZnO	Sol–gel	As(iii) concentration = 5 ppm	Mercury lamp 350 nm	100% in 120 minutes	67
		Catalyst load = 0.5 g L^−1^			
		pH = 8			
ZnO	Dip-coating	As(iii) concentration = 3 ppm	2 UV-C 15 watts lamps 265 nm	100% in 60 minutes	68
		pH = 4			
ZnO/TiO_2_	Coprecipitation	As(iii) concentration = 1 ppm	UV 8 W lamp 365 nm and sunlight	90% in 120 minutes	69
CuO/ZnO	Mechanical milling	As(iii) concentration = 30 ppm	Black light of 352 nm	100% in 150 minutes	70
		Catalyst load = 1 g L^−1^			
		pH = 7			
Cu–ZnO	Coprecipitation	As(iii) concentration = 5 ppm	LED strip 10 W, 400–600 nm	100% in 240 minutes	71
		Catalyst load = 3 g L^−1^			
Cu–ZnO/polystyrene pellets	Solvent casting	As(iii) concentration = 5 ppm	LED strip 10 W, 400–600 nm	100% in 150 minutes	72
		Catalyst load = 25 g L^−1^			
Cu–ZnO	Chemical synthesis	As(iii) concentration = 30 ppm	Blue lamp, 460 nm	90% in 6 hours	73
		Catalyst load = 8.3 g L^−1^			

**Fig. 8:**
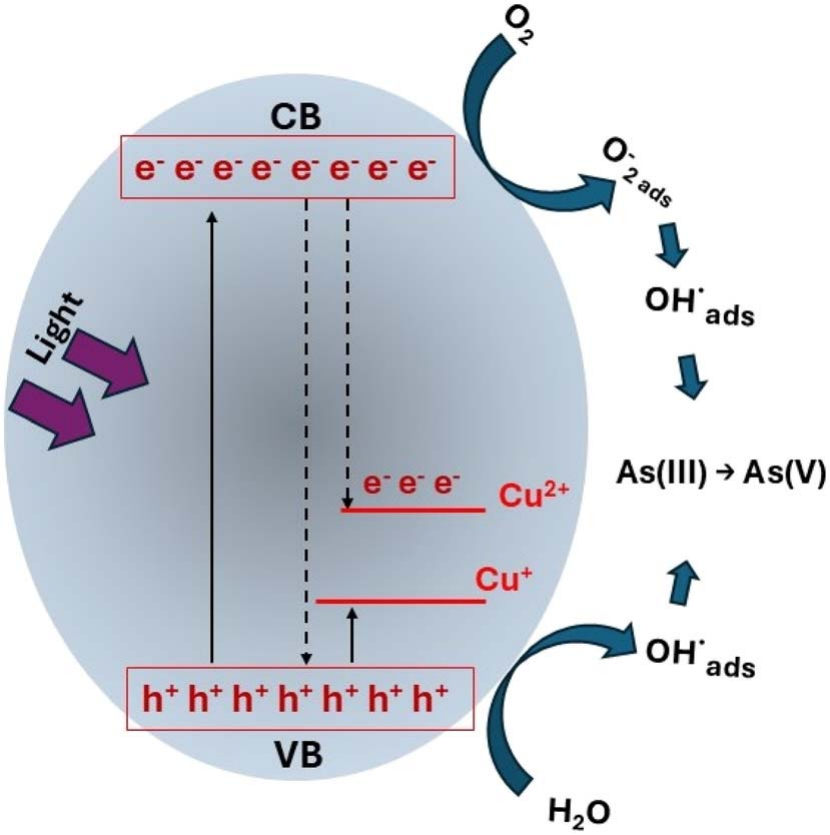
Representation of Cu-doped ZnO by Gyrdasova and co-workers and its effect on As(iii) photooxidation.^73^

However, due to its large band gap, ZnO's photoresponse range is limited to ultraviolet light, which accounts for only 5% of solar radiation, resulting in poor solar energy utilization and low quantum efficiency.^74,75^ Furthermore, like all photocatalysts, ZnO exhibits the problem of rapid electron/hole pair recombination, which decreases its photocatalytic activity.^76^ Therefore, considerable efforts have been devoted to optimizing ZnO's photocatalytic activity, including cocatalyst loading, heteroatom doping, and heterojunction construction.^77,78^ Arabnezhad and collaborators obtained ZnO/TiO_2_ heterojunctions at different concentrations through the coprecipitation process, these heterojunctions were applied for the oxidation of As(iii) under both UV and solar irradiation.^69^ The efficiency of As(iii) oxidation depended on the type of irradiation and the ZnO/TiO_2_ ratio. The highest As(iii) oxidation efficiency (90% in 120 min) was achieved with the 90 : 10 ratio sample under ultraviolet light; while under sunlight irradiation, the highest efficiency was achieved with the 50 : 50 ratio sample. CuO is one of the most reported materials that forms heterojunctions with ZnO for the photocatalytic applications, this is due to the synergistic effects between the two oxides where the visible light absorption capacity of copper oxide is combined with the electron–hole separation efficiency of zinc oxide.^79^ Samad and collaborators obtained CuO/ZnO mixtures at different concentrations (5, 10, 20 and 50% CuO) using the mechanical milling technique from commercial oxides. The photocatalytic results showed that the CuO/ZnO mixtures outperformed the oxides alone under UV irradiation (352 nm); the best material was the 20% CuO material, which completely oxidized a 30 ppm As(iii) solution in 150 min.^70^ Regarding heteroatom doping, copper is an effective acceptor impurity that affects the electronic band structure of ZnO.^80^ Vaiano and collaborators synthesized Cu-doped ZnO at different concentrations using the coprecipitation technique, managing to oxidize As(iii) with visible light, with the catalyst with a doping of 1.08 mol% being the best, reaching 100% of As(iii) oxidation in 240 minutes.^71^ In subsequent work, the same authors successfully prepared a supported Cu-doped ZnO material on polystyrene pellets for possible large-scale application.^72^ Their experiments showed that a 5% by weight Cu-doped ZnO pellet supported on the pellets showed the best performance, achieving 100% oxidation of a 5 ppm As(iii) solution in 150 minutes under visible irradiation. In the same direction, Gyrdasova and co-workers synthesized Cu-doped ZnO by a chemical route in glycol-formate medium. The materials showed adsorption and photocatalytic properties in visible light for the oxidation of As(iii), with the material doped with 0.15 mol% of Cu being the best, oxidizing up to 90% of As(iii) in 6 hours. The authors attributed the efficiency under visible light to the incorporation of copper, which effectively reduces the ZnO band gap by introducing additional energy levels into its electronic structure, which causes an expansion of the zinc oxide's photoactivity into the visible range (Fig. 8). Furthermore, the Cu^+^/Cu^2+^ copper ions also contribute to the exciton pair separation.^73^

### 4.3. Iron based photocatalysts

In recent years, photocatalysts based on iron compounds have shown remarkable potential in the oxidation of As(iii) to As(v) in aqueous systems, thanks to their low cost, high availability, and environmental compatibility. Materials such as oxides (Fe_2_O_3_, Fe_3_O_4_) and iron oxyhydroxides (FeOOH) have been studied for their ability to generate reactive oxygen species (ROS) under irradiation, facilitating effective redox reactions. The oxidation of As(iii) to As(v) using iron-based photocatalysts is mainly carried out through a heterogeneous photo-Fenton process, in which irradiation with light (UV or visible) excites the material, generating electron–hole pairs. These charge carriers induce the formation of reactive oxygen species, such as hydroxyl radicals (˙OH) and superoxide (˙O_2_^−^), responsible for the oxidation of As(iii). Additionally, in the presence of H_2_O_2_, Fenton-type reactions are activated on the surface of the catalyst, increasing the production of ˙OH. The highly toxic and mobile As(iii) is thus transformed into As(v), a less toxic species that is more easily retained by adsorption. This mechanism can be optimized by doping the material or forming heterostructures that improve photoinduced efficiency. Yanmei Li *et al.* studied the effect of a heterogeneous photo-Fenton catalyst composed of Mn FeOOH nanoparticles supported on a highly porous carbonized aerogel. Doping with Mn accelerated the regeneration of Fe^2+^ and promoted the activation of H_2_O_2_, generating 2.4 times more hydroxyl radicals (˙OH) compared to pure FeOOH. This improvement enabled the complete degradation of As(iii) in just 25 minutes under irradiation, while maintaining more than 80% of its capacity after five cycles of use. The system optimizes electron transfer at the interface, offering a practical and durable strategy for treating contaminated water.^81^ For their part, Lucía *et al.* synthesized Fe–Cu magnetic nanocomposites prepared by pyrolysis using carbon sources derived from urban waste, which were irradiated continuously, exhibiting a substantial reduction in As(iii) of between 50% and 71%. These results demonstrate significant photocatalytic activity even without the need for intense UV light. The results imply that the pyrolysis temperature during synthesis allowed the predominant oxidation pathway to be modulated—either through valence band gaps or hydroxyl radicals—opening up the possibility of optimizing the design of photocatalysts for efficient arsenic remediation in water.^82^ Jinglin *et al.* developed iron oxide nanocomposites (α-FeOOH, α-Fe_2_O_3_, and Fe_3_O_4_) supported on hydrothermal carbon to enhance the photo-Fenton-like oxidation of As(III) in the presence of oxalate. In systems with oxalate (0.5 mmol L^−1^), saturated oxygen, and pH 5, both α-FeOOH and α-Fe_2_O_3_ achieved complete conversion of As(iii) to As(v) in 15 and 20 min, respectively. The mononuclear Fe^3+^ oxalate complex exhibited high photosensitivity, accelerating the regeneration of Fe^2+^ and the generation of oxidizing radicals that are responsible for its photocatalytic efficiency. This approach offers a robust and efficient route for arsenic remediation in water using iron-based materials and organic chelating agents.^83^ Dashi Lee *et al.* synthesized a core–shell photocatalyst (ZnFe_2_O_4_@PANI) that activates sulfite (SO_3_^2−^) under visible light, generating dominant oxidizing species such as sulfate radicals (SO_4_˙^−^) and hydroxyl radicals (˙OH). This activation enabled the complete oxidation of As(iii) in just 60 minutes, compared to only ∼60% efficiency with uncoated ZnFe_2_O_4_. The system showed high stability against pH (3–10) and easy magnetic recovery, with minimal Fe leaching. The mechanism involved the Fe(iii) oxalate/Fe(iv) complex as a key intermediate, accelerating radical generation and promoting Fe(ii) regeneration.^84^

**Fig. 9:**
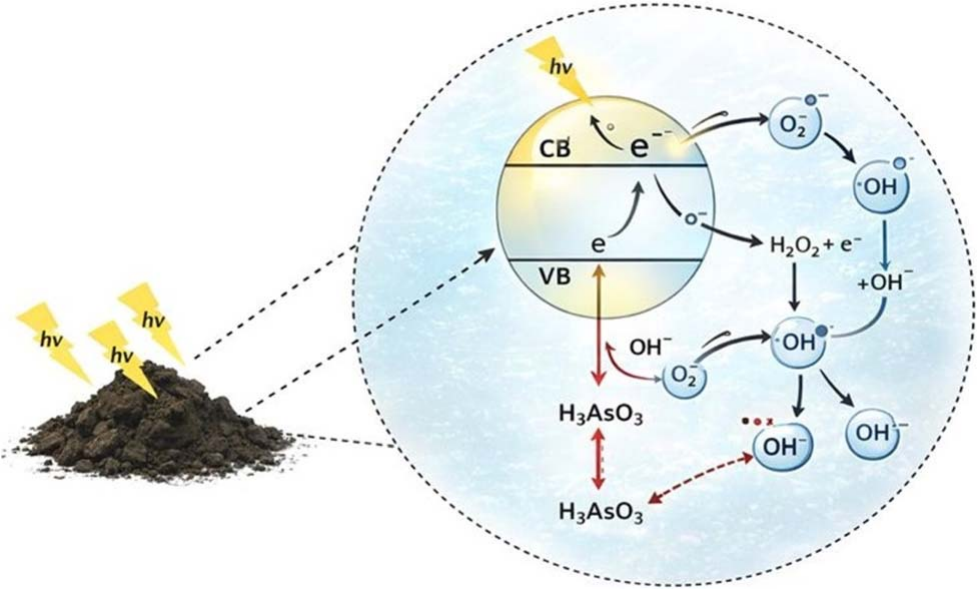
Synergistic mechanism of As(iii) oxidation–adsorption by TiO_2_ and zero-valent iron.

According to M. J. *et al.*, the application of systems based on TiO_2_, zero-valent iron (ZVI), and their combination under UV irradiation constitutes a synergistic strategy for the removal of As(iii) in the aqueous phase. This approach integrates TiO_2_-induced photocatalytic oxidation with the adsorption of As(v) on corrosion products generated from ZVI, resulting in highly efficient removal of the pollutant. Fig. 9 shows a representative scheme adapted from their research.^85^

### 4.4. Carbon-based photocatalysts

Carbon-based nanomaterials and nanocomposites address the need for a potential photocatalyst for large-scale industrial use, as it is economical, environmentally friendly, and sustainable. Carbon has been widely used as support in various forms in different applications thanks to its excellent electronic, electrochemical, and physicochemical properties.^86^ Carbon-based nanostructures, such as graphite, diamond, graphene, carbon nanotubes (CNTs), fullerene, and carbon nitride (g-C_3_N_4_), are attractive in the field of photocatalysis for various reactions. Among them, graphene oxide is the most promising, with its two-dimensional nanostructure with sp_2_ hybridization and zero band gap, featuring a layered structure of carbon atoms arranged in an atomic-thick honeycomb. The graphene oxide nanostructure features the largest specific surface area, relatively high electron mobility, excellent thermal and electrical conductivity, and optical transparency.^87^ Moon and collaborators dispersed TiO_2_ powder (P25) on graphene oxide by the pH-induced precipitation method, synthesizing composites at different proportions of TiO_2_ on graphene oxide.^88^ Photooxidation experiments of As(iii) under a 300 W light source with a 10 cm IR filter and a cut-off filter (*λ* ≥ 320 nm) showed that when the TiO_2_ concentration was equal to or greater than 0.6% by weight, complete oxidation was achieved in 30 minutes. Finally, the authors compared the photocatalytic properties of the TiO_2_/rGO composite against platinum-supported TiO_2_ (Pt/TiO_2_) composites, demonstrating similar behavior, although the time to achieve 100% oxidation of As(iii) is 20 minutes slower, the much lower cost of the TiO_2_/rGO composite makes it a very interesting alternative. In another work, Zhu and collaborators prepared mixed oxide composites, obtained from hydrotalcites, with different amounts of reduced graphene oxide through the hydrothermal process. The materials obtained were evaluated in the simultaneous processes of As(iii) oxidation and paracetamol degradation in water, bringing the experiments closer to real conditions where As(iii) is mixed with other contaminants.^89^ Experiments under irradiation of a 500 W lamp with a 300 nm cut-off filter showed that the composite synthesized with 30 mg of graphene oxide (named CLDH/rGO30) obtained the best performance in paracetamol degradation and achieved 99% removal of As(iii) within 10 minutes. Zhang and coworkers synthesized a FeOOH/graphene oxide heterostructure using a combination of the Hummers' method and chemical precipitation. The authors used different amounts of Fe^3+^ during the synthesis to vary the amount of the metallic phase in the material. The total amounts of As(v) formed increased in the FeGO systems with increasing FeOOH loadings, with the best material at 0.5 mM Fe^3+^ reaching an efficiency of 75% under irradiation of a 500 W Xe lamp (light density 32 mW cm^−2^).^90^ Zhuanhong Lu and colleagues formed van der Waals heterostructures (vdWHs) by obtaining pyrrolic N, pyridinic N, and graphitic N by assembling indole, quinoline, and indolizine with graphene oxide.^91^ Pyrrolic nitrogen showed the best catalytic activity in vdWH reaching 60.22% As(v) after 15 h irradiation with Xe lamps (500 W) located at 15 cm from the experiment, while pyridinic nitrogen was the worst reaching only 16.68%. The superior photocatalytic performance was attributed to its more efficient electron injection and stronger photocurrent response.

g-C_3_N_4_ materials represent a potential metal-free semiconductor photocatalyst in diverse applications, thanks to their simple synthesis methods, their suitable electronic band structures (2.7 eV), and their excellent physicochemical stability. Furthermore, as a nontoxic semiconductor catalyst, g-C_3_N_4_ offers great potential in environmental restoration and photocatalytic processes.^92,93^ Jong-Gook Kim and co-workers synthesized g-C_3_N_4_ from urea using the thermal polycondensation route and showed that the material is active for the simultaneous oxidation of methyl orange and As(iii) under 365 nm UV light.^94^ The authors increased the reaction efficiency by adding potassium persulfate to the reaction for the generation of sulfate radicals, achieving an 80% oxidation of As(iii) when it is alone in the solution and 50% when mixed with the methyl orange. Zhao Wang and colleagues obtained materials based on g-C_3_N_4_ and PMDA at different ratios, and were evaluated in the simultaneous reactions of As(iii) oxidation and Cr(vi) reduction under a 300 W Xenon lamp with a cutoff filter of 420 nm.^95^ The best material was synthesized with a 1 : 2 ratio of g-C_3_N_4_ : PMDA, achieving total oxidation of a 7.5 ppm As(iii) solution in 100 minutes. Chunli Wang *et al.* took advantage of the 2D morphology of g-C_3_N_4_ and synthesized a 2D/2D g-C_3_N_4_/bentonite composite *via* the thermal polycondensation route. Their experiments showed that the ratio of 10% bentonite over g-C_3_N_4_ has a higher oxidation of As(iii) reaching 100% in 3 hours under irradiation of a 300-W xenon lamp.^96^ The authors investigated the influence of composite's synthesis temperature on the photocatalytic oxidation properties of As(iii); they evaluated three temperatures: 450, 550, and 650 °C, and found 550 °C to be the optimum. Hanyu Liu and colleagues synthesized a heterojunction of carbon-doped TiO_2_ and nitrogen-deficient g-C_3_N_4_ by a combination of thermal polycondensation, acid treatment, and chemical coprecipitation processes.^97^ The synthesized compound not only has good adsorption capacity of As(iii) in the dark reaction stage but also can quickly complete the whole photocatalytic process within 12 minutes after the photoreaction, while adsorbing As(v) generated by the oxidation of As(iii). Debanjali Dey and his collaborators succeeded in synthesizing a La–Al_2_O_3_/gC_3_N_4_ heterojunction in the form of an agarose-based aerogel using thermal polycondensation, coprecipitation, and freeze-casting techniques. The heterojunction showed bifunctional ability to adsorb and photooxidize As(iii) under a UV (365 nm) and visible light. Heterojunction demonstrated a higher removal rate of 66.5% under ultraviolet light, 57% under visible light, and 42% in the dark.^98^ Guotao Hu and co-workers focused on improving the reactivity of the surfactant sites of g-gC_3_N_4_ by introducing COOH to modify the edge sites. The modifications to g-gC_3_N_4_ were carried out using formaldehyde during the thermal polycondensation process at different temperatures (450, 500, 550 and 600 °C). The results showed that the modified g-C_3_N_4_ obtained at 500 °C (designated ECCN-500) exhibited a remarkable photocatalytic oxidation rate of 93% for trivalent arsenic, four times higher than that of C_3_N_4_. The authors attribute this to the presence of COOH units at the edge, which significantly disrupts the charge distribution in the melem units.^99^Table 2 summarizes recent advances in carbon-based materials applied to the photocatalytic oxidation of As(iii). In general, the studies highlight the role of carbon-based structures in creating efficient and sustainable photocatalytic systems for arsenic remediation.

**Table 2:** Comparison of carbon based photocatalysts for As(iii) oxidation: synthesis methods, experimental conditions and efficiency

Catalyst	Synthesis method	Experimental conditions	Irradiation	As(iii) oxidation	Ref.
TiO_2_/rGO	Chemical precipitation	As(iii) concentration = 37 ppm	300 W source with a 320 nm cut-off filter	100% in 30 minutes	88
		Catalyst load = 0.5 g L^−1^			
		pH = 3			
Calcined LDH/rGO	Hydrothermal	As(iii) concentration = 37 ppm	A 500 W lamp with a 300 nm cut-off filter	99% in 10 minutes	89
		Catalyst load = 0.5 g L^−1^			
		pH = 3			
FeOOH/GO	Chemical precipitation	As(iii) concentration = 7.5 ppm	500 W Xe lamp light density of 32 mW cm^−2^	75% in 12 hours	90
		Catalyst load = 0.3 g L^−1^			
		pH = 6			
Amine derivative/GO	Hummers and ultrasonic	As(iii) concentration = 750 ppm	500 W Xe lamp	60% in 15 hours	91
		Catalyst load = 10 g L^−1^			
g-C_3_N_4_	Polycondensation	As(iii) concentration = 100 ppm	60 W UV lamp of 365 nm	80% in 6 hours	94
		Catalyst load = 5 g L^−1^			
g-C_3_N_4_/PMDA	Polycondensation/calcination	As(iii) concentration = 7.5 ppm	300 W Xenon lamp with a 420 nm cutoff filter	100% in 100 minutes	95
		Catalyst load = 1 g L^−1^			
g-C_3_N_4_/bentonite	Polycondensation	As(iii) concentration = 10 ppm	300 W Xenon lamp	100% in 3 hours	96
		Catalyst load = 0.2 g L^−1^			
		pH = 8.5			
(C/TiO_2_@ND-C3N4)	Polycondensation/acid treatment/chemical coprecipitation	As(iii) concentration = 10 ppm	300 W Xenon lamp with a 420 nm cutoff filter	95% in 12 minutes	97
		Catalyst load = 0.5 g L^−1^			
		pH = 8.5			
La-doped Al_2_O_3_/g-C_3_N_4_/agarose aerogel	Polycondensation/coprecipitation/freeze-casting techniques	As(iii) concentration = 10 ppm	450 W mercury lamp (365 nm) and visible light	66% in 3 hours	98
		Catalyst load = 1 g L^−1^			
		pH = 3			
COOH-g-C_3_N_4_	Polycondensation	As(iii) concentration = 10 ppm	300 W Xenon lamp with a 400 nm cutoff filter	93% in 90 minutes	99
		Catalyst load = 0.5 g L^−1^			
		pH = 3			

## 5. Mechanisms for the arsenic(iii) oxidation by photocatalysis

The photocatalytic oxidation of As(iii) into As(v) represents a fundamental process in water remediation, as it transforms a highly toxic and mobile species into a less toxic and more easily removable form. Several studies have demonstrated that photocatalysis, primarily using materials such as semiconductors and mixed oxides, enables a rapid and efficient conversion of As(iii) to As(v) under UV or visible irradiation, facilitating their further elimination through adsorption.^100^ Understanding the mechanism involved in this transformation is crucial for optimizing the treatment process and developing more effective new photocatalytic materials. In this context, reactive species such as hydroxyl radicals (˙OH), and superoxide (O_2_˙^−^), as well as the participation of photo-induced holes (h_BV_^+^), have been identified as key agents in As(iii) oxidation, depending on the system conditions and the nature of the photocatalyst.^101,102^ The reactions involved in the oxidation of As(iii) to As(v) have been extensively investigated by several authors.^103–108^ It has been established that this process proceeds through monoelectronic stages in which arsenic passes through an intermediate As(iv) state before reaching its pentavalent form.^96^ The progression of these stages is mainly facilitated by highly oxidizing species, such as hydroxyl radicals (˙OH), superoxide species (O_2_˙−), and photogenerated holes (h_BV_^+^), with hydroxyl radicals and superoxides being the predominant oxidizing agents.^109,110^ The proposed mechanism for this reaction is influenced by several factors, including the solution's pH, the semiconductor's nature, and the characteristics of the radiation employed.^111^ The process begins after the absorption of photons (*hν*) with energy greater than the semiconductor's band gap. Subsequently, an electron in the valence band (VB) will be excited and propelled to the conduction band (CB), leaving a positively charged hole in the valence band. The photogenerated e_BC_^−^/h_BV_^+^ pair will migrate to the surface of the semiconductor. In the case of TiO_2_, these pairs will be trapped by the titanol groups on the surface:

1 TiO_2_ + *hν* → TiO_2_(h_BV_^+^ + e_CB_^−^)

2
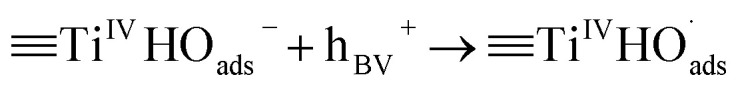

3 ≡Ti^IV^HO_ads_^−^ + e_CB_^−^ ↔ ≡Ti^III^OH

Electrons and holes that did not undergo recombination processes may react with oxygen dissolved in the medium or adsorbed water molecules. Under aerobic conditions, oxygen serves as the primary electron acceptor, generating superoxide radicals. Superoxide can react by disproportionation into H_2_O_2_ and O_2_, or it can be further reduced on the surface, also producing H_2_O_2_. While adsorbed H_2_O_2_ can react with an electron or a hole, holes can also oxidize adsorbed hydroxide, forming hydroxyl radicals.

4 O_2_ + e_CB_^−^ → O_2_˙^−^

5
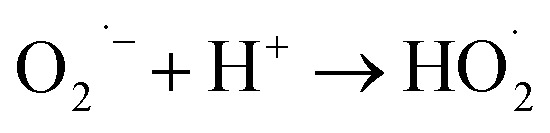

6 O_2_˙^−^ + e_CB_^−^ + H^+^ → H_2_O_2_

7
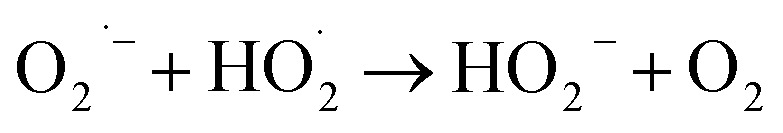
8
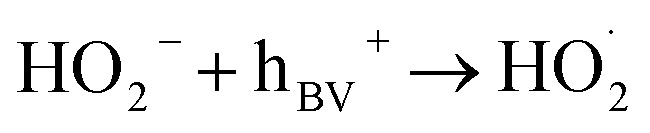
9
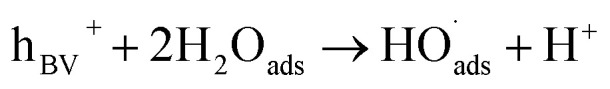

10 H_2_O_2_ + e_CB_^−^ → HO˙ + HO^−^

The species generated above will participate in redox reactions if they are thermodynamically favorable. As(iii) has a high thermodynamic driving force to oxidize to As(v) in UV/TiO_2_ systems across all pH ranges. The species h_BV_^+^, H_2_O_2_, O_2_˙^−^*y*HO˙ can oxidize As(iii) species to As(iv) species. As(iv) can exist as four different species, As^IV^–(OH)_4_, As^IV^(OH)_3_O^−^, HAs^IV^O_3_^−^*y*As^IV^O_3_^2−^, depending on the pH. The various As(iv) species disappear in second-order reactions to generate As(v) and As(iii).

11 As^III^(OH)_3_ + h_BV_^+^ → As^IV^(OH)_4_

12 As^III^(OH)_3_ + H_2_O_2_ → As^IV^(OH)_4_ + 2HO^−^

13 As^III^(OH)_3_ + O_2_˙^−^ + H_2_O + H^+^ → As^IV^(OH)_4_ + H_2_O_2_

14
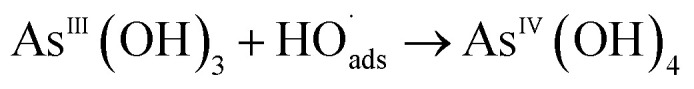

15 As^III^(OH)_4_ ↔ HAs^IV^O_3_^−^ + H_2_O + H^+^

16 As^III^(OH)_4_ ↔ As(OH)_3_O^−^ + H^+^

17 HAs^IV^O_3_^−^ ↔ As^IV^O_3_^2−^ + H^+^

18 As(OH)_3_O^−^ ↔ HAs^IV^O_3_^−^ + H_2_O

19 As^IV^(OH)_4_ → As(iii) + As(v)

20 As^IV^(OH)_4_ + HAs^IV^O_3_^−^ → As(iii) + As(v)

21 2HAs^IV^O_3_^−^ → As(iii) + As(v)

22 2As^IV^O_3_^2−^ → As(iii) + As(v)

23 2HAs^IV^O_3_^−^ + As^IV^O_3_^2−^ → As(iii) + As(v)

Despite the application of different light intensities and other experimental conditions, the oxidation of As(iii) to As(v) in batch systems is generally completed in time scales of <30–100 min. Some authors have proposed their own mechanisms under their operating conditions. For example, Pin Li *et al.* evaluated the oxidative behavior of As(iii) in the presence of birnessite, comparing photolysis and photocatalysis conditions under visible light. Under photolysis conditions, partial conversion of As(iii) to As(v) was observed, mediated by the oxidizing action of surface Mn(iv), which is reduced to Mn(ii) and subsequently precipitates as MnOOH. This secondary phase involves active sites on the birnessite, which limit the process efficiency and achieve only ∼60% conversion after 360 minutes at pH 5. In contrast, under visible light irradiation, birnessite exhibited significantly higher photocatalytic activity, achieving conversions of nearly 100% within the same time interval, in a pH range of 5 to 8. Mechanistic analysis revealed that the photogeneration of valence band holes (h^+^) and superoxide radicals (˙O_2_^−^) on the mineral surface is a key reactive species for oxidation. Unlike the photolysis process, the formation of MnOOH does not significantly block active sites during photocatalysis, allowing sustained oxidation. It is concluded that photogenerated holes act as the primary oxidizing agent, facilitating direct electron transfer from As(iii), while ˙O_2_^−^ radicals act as secondary co-agents, enhancing the efficiency of the system under visible light conditions.^112^ Wenke Zhu *et al.* demonstrated that simultaneous doping of TiO_2_ with Sn and N extends spectral absorption toward the visible spectrum through the formation of O–Ti–N bonds, which introduce intermediate electronic levels that favor the efficient generation and separation of electron–hole pairs. This co-doping results in improved charge transfer and reduced electron recombination, leading to increased photocatalytic activity under visible irradiation. The proposed mechanism involves the synergistic action of three reactive species generated on the surface of the photocatalyst: holes (h_BV_^+^), which can directly oxidize As(iii); hydroxyl radicals (˙OH), formed from water or OH^−^ ions, which contribute as secondary oxidants; and superoxide radicals (O_2_˙^−^), generated by the reduction of oxygen in the conduction band. Tests with radical scavengers confirm that ˙OH radicals are primarily responsible for oxidation, while superoxides play a complementary but relevant role in the process.^113^ Laura Chianese *et al.* investigated the primary reactive species involved in the oxidation of As(iii). Experiments were conducted with radical scavengers, which indicated that the dominant species responsible for oxidizing As(iii) are the superoxide radical (O_2_˙^−^) and free electrons (e^−^). Holes (h^+^) may also participate, but in these studies, their contribution is minor or secondary compared to O_2_˙^−^ and free electrons (e^−^). The reaction mechanism indicates that electrons in the conduction band reduce dissolved O_2_, generating O_2_˙^−^. Subsequently, this radical attacks As(iii), converting it to As(v). Electrons can interact directly with As(iii) or influence additional redox pathways. The effect of doping and structure in doped versions, such as BiFeO_3_ with gadolinium (Gd), improves charge separation and increases the production of free O_2_˙^−^ and e^−^, significantly increasing oxidation efficiency under visible light.^114^

## 6. Limitations and future research

One of the main challenges in the field of photocatalytic oxidation, not only for As(iii) but also for various organic pollutants, is the limited utilization of the solar spectrum by photocatalysts. Titanium dioxide (TiO_2_) remains the most studied and applied semiconductor material due to its chemical stability, low cost, and high oxidative power. However, its bandgap value of around 3 eV (for the anatase phase)^115^ restricts photoactivation to the ultraviolet (UV) region of the spectrum, which represents only 3% to 5% of the total solar radiation reaching the Earth's surface.^116^ This significantly limits the efficiency of TiO_2_-based systems under natural sunlight, limiting their practical application in large-scale or field-based water treatment systems. To reduce the band gap of TiO_2_, numerous strategies have been proposed, such as elemental doping like carbon, sulfur, nitrogen and fluor,^117–119^ the incorporation of metal ions^120^ (*e.g.*, Fe^3+^, Cu^2+^), and the formation of heterojunctions with narrow-band gap semiconductors such as tungsten trioxide (WO_3_),^121^ bismuth vanadate (BiVO_4_),^122^ or cadmium sulfide (CdS).^123^ These modifications aim to shift the absorption edge of the photocatalyst toward the visible light region (400–700 nm), which constitutes almost 43% of the solar spectrum, thereby improving sunlight harvesting. Despite these advances and research efforts, many of these modified systems present several drawbacks, primarily the rapid recombination of photogenerated electron–hole pairs. This recombination process occurs in nanoseconds and significantly limits the availability of charge carriers for redox reactions, ultimately reducing photocatalytic efficiency. Furthermore, some doped or hybrid materials may exhibit low long-term stability (compared to pure TiO_2_), photocorrosion, or toxicity issues associated with certain dopants or secondary phases, further complicating their application in environmental remediation.

A major limitation in the application of photocatalysis, particularly for water treatment, lies in the stability and reusability of photocatalytic materials. Although several metal oxide semiconductors have demonstrated high photocatalytic activity under UV or visible light, many of them, such as ZnO, suffer from photocorrosion, especially under acidic conditions.^124^ This not only reduces long-term photocatalytic efficiency but also poses environmental and health risks due to potential secondary contamination by dissolved metal species. Furthermore, one of the main operational challenges in real-world applications of large-scale photocatalysis is the recovery and reuse of nanoparticle-based photocatalysts. While their nanometric size offers significant advantages due to their high surface area and reactivity, it also makes it difficult to separate from treated effluents using conventional filtration or sedimentation techniques. This complication can increase operational costs and hamper regulatory compliance if nanoparticles remain in the effluent. To address this issue, various strategies have been proposed, such as the immobilization of photocatalysts on inert or functional supports, such as activated carbon, silica, or magnetic nanoparticles (*e.g.*, Fe_3_O_4_).^125^ These composite systems facilitate catalyst separation and reuse *via* magnetic recovery or filtration. Another strategy is the fabrication of coatings supporting photocatalytic nanoparticles or photocatalytic oxides.^126^ However, such immobilization typically leads to a reduction in catalytic activity due to limited exposure of the active sites, reduced surface area, or mass transfer limitations.

Another important limitation in the large-scale application of photocatalysis for As(iii) oxidation lies in the chemical composition of real-world water bodies. Unlike controlled laboratory conditions where ultrapure water and pure reagents are used, natural and wastewater sources often contain diverse anions such as phosphate (PO_4_^3−^), sulfate (SO_4_^2−^), bicarbonate (HCO_3_^−^), and chloride (Cl^−^); and dissolved organic matter that can significantly affect the efficiency of photocatalytic processes.^127^ These anions and natural organic matter (NOM) can act as competing species that interfere with the generation and activity of reactive oxygen species (ROS), including hydroxyl radicals (˙OH) and superoxide radicals (˙O_2_^−^).^128^ Overcoming these interferences requires the development of more selective photocatalysts (either by modifying the photocatalysts to have a high surface specificity for As(iii) or by modifying the external conditions), greater resistance to fouling, and sustained activity in the presence of competing species.^129^ Similarly, the implementation of hybrid adsorption/photocatalysis strategies in the processes can be a way to reduce the impact of various anions on the photocatalysts (Table 3).

**Table 3:** Literature summary table for different photocatalysts using for As(iii) in As(v) photocatalytic oxidation

Catalyst	Characteristics/advantages	Limitations/disadvantages	As(iii) removal	Ref.
Manganese oxides	Structure and crystallinity variables, obtaining materials with different valences (Mn^2+^, Mn^3+^, Mn^4+^) that facilitate redox reactions	Manganese oxides can suffer partial dissolution or structural transformation during the process, which reduces their activity and useful life	96–100%	130–132
	Significant adsorption capacity through arsenic species, due to the presence of hydroxyl groups and surface hydroxyls, and variable charge with the pH	Their efficiency decreases in an acidic medium or a very alkaline medium, since their potential redox pair (Mn(iv) → Mn(ii)) and the arsenic speciation depend highly on pH		
	MnOx can act simultaneously as adsorbents and oxidants, reducing the need to add additional reagents	It has a high rate of recombination of charge pairs, which reduces the quantum efficiency		
Titanium dioxide	High superficial area (especially in anatase phase and nanometric morphologies)	Absorption limit to the UV spectrum	99–100%	133–135
	Good adsorption capacity of oxyanions like As(iii) and As(v), depending on pH and superficial charge	The presence of competing ions (such as phosphates, silicates, carbonates, or sulfates) can inhibit As adsorption and decrease photocatalytic activity		
	It can be modified by doping (metals, non-metals) or composites (TiO_2_–Fe, TiO_2_–MnO_2_, TiO_2_–C, TiO_2_–g-C_3_N_4_, *etc.*) to extend the spectral response to the visible range and improve electron–hole separation	The accumulation of compounds on the surface of TiO_2_ occasionally blocks active sites, decreasing its photocatalytic activity		
Zinc oxides	Is inexpensive, non-toxic, and highly available	Under UV irradiation, ZnO can undergo autodissemination (Zn^2+^ released into the environment), which degrades the structure of the catalyst and can generate secondary contamination by zinc	95–98%	136 and 137
	It can be synthesized into nanoparticles, nanorods, nanoflowers, and thin films, allowing for adjustments in morphology to optimize contaminant adsorption and expose active sites	Their large band gap restricts absorption, primarily in the UV region		
	It can be synthesized through economic methods and at low temperatures, such as precipitation, sol–gel, or hydrothermal synthesis, allowing for large-scale production	In acid conditions, the ZnO tends to dissolve forming Zn^2+^ ions, while in alkaline media, it tends to precipitate as Zn(OH)_2_		
Iron-based materials	Some oxides, like hematite (α-Fe_2_O_3_), can absorb up to 40% of the solar spectrum	Many iron oxides, such as hematite, exhibit electronic transitions and low charge mobility, which limits their efficient utilization of visible radiation	99–100%	138–140
	In phases like Fe_3_O_4_ (magnetite), the material can be recovered easily through magnetic separation	The formation of As(v) layers or iron hydroxyls on the surface can block the active sites		
	Iron presents multiple oxidation states (Fe^2+^/Fe^3+^), which allows it to participate in electron transfer processes, as well as in the efficient adsorption of As(iii) and As(v) on its surface	Poor charge separation and low quantum efficiency		
Carbon-based materials	Large surface area and porosity	Low intrinsic photocatalytic activity: does not generate effective electron–hole pairs under irradiation		141–143
	It can facilitate the rapid electron transferences, acting as “bridges” or charge collectors that reduce electron–hole recombination in composite systems	Dependency on coupled materials, which may result in a possible recombination of charges if there is no good coupling		
	They are highly resistant to chemical and thermal degradation, enabling them to maintain their structure and performance even after multiple cycles of use	The redox potential of some carbon-based materials is not always sufficient for complete As(iii) oxidation, especially under visible irradiation		

## 7. Perspectives

As presented in this review and as reported in the literature, the use of photocatalytic processes represents a promising and environmentally friendly strategy for arsenic remediation, particularly due to their potential to oxidize the highly toxic and mobile As(iii) species into the less hazardous and more readily adsorbable As(v) form. Despite continuous progress in the development of photocatalytic materials with greater efficiency and selectivity, several challenges remain before this technology can be implemented on a large scale or in real-world systems.

Future research could focus on the development of multifunctional materials that can function for both adsorption and photocatalytic oxidation processes. Such multifunctional systems could ensure the simultaneous capture and transformation of As(iii), minimizing its release into water. The design and development of these materials require a set of processes such as the control of surface chemistry, porosity, and interfacial interactions.

Similarly, future research should focus on designing photocatalysts with greater light-harvesting capacity, including robust activity in visible light, while also exhibiting efficient charge carrier separation. This can be achieved through the implementation of heterostructures/heterojunctions, dopants, and cocatalysts, which play a crucial role in promoting redox reactions and potentially improving the overall photocatalytic efficiency for arsenic oxidation.

Research should also consider sustainable and low-cost synthesis methods that facilitate scalability, enabling viable industrial and environmental applications. Within these methodologies, it is essential to incorporate the principles of green chemistry through the use of benign solvents, renewable precursors, and energy-efficient synthesis routes to minimize the environmental impact during material production. Similarly, currently used/reported manufacturing techniques, such as sol–gel, hydrothermal, or coprecipitation methods, must be optimized to obtain photocatalysts on a large scale without compromising their structural integrity or photocatalytic performance. This optimization of synthesis processes to scalable and economically accessible routes is essential to promote the practical application of photocatalytic arsenic oxidation technologies in real water treatment systems. Another major problem that must be addressed is that most studies have been conducted in controlled systems with synthetic solutions, which do not reflect the chemical complexity of real water or industrial effluents containing competing ions, organic matter, and fluctuating pH conditions. It is vital that future research evaluate the performance, reuse, and long-term stability of photocatalysts under realistic environmental conditions.

Finally, a deeper understanding of the photocatalytic oxidation of arsenic is still needed, as understanding the role of reactive oxygen species and surface intermediates can aid in the design of next generation photocatalysts. Combining experimental observations with computational modeling and advanced characterization techniques will be key to optimizing photocatalytic oxidation processes. In this regard, combining sciences such as materials science, environmental chemistry, and process engineering will be crucial to elevating the photocatalytic oxidation of As(iii) from a promising laboratory process to a viable and sustainable technology for treating contaminated water.

## 8. Conclusions

Heterogeneous photocatalysis has become a quite promising strategy for the oxidation of As(iii) to As(v), allowing its subsequent removal by conventional separation processes. As summarized throughout this work, semiconductor materials, such as TiO_2_, ZnO, WO_3_, Fe-based oxides and carbon-based photocatalysts, have shown rapid and efficient oxidation under both ultraviolet and visible irradiation, achieving conversion rates greater than 90% on time scales ranging from a few minutes to a few hours. Recent advances in materials engineering, such as doping with both metallic and non-metallic elements, modulation of oxygen vacancies, and development of heterojunctions (*e.g.*, TiO_2_/WO_3_, ZnO/CuO, FeOOH-carbon systems), have expanded light absorption and improved charge carrier separation, significantly improving photocatalytic performance. Carbon-based materials, such as those derived from graphene and g-C_3_N_4_, have gained notable relevance as both sustainable and low-cost alternatives due to their high conductivity, stable structure and ability to act as charge mediators. Similarly, the emergence of multifunctional photocatalysts that combine oxidation and adsorption capabilities highlights a new direction toward more integrated remediation systems.

Despite these advances, the practical implementation of photocatalytic arsenic oxidation remains a challenge due to various complications, some of which are inherent to photocatalysts, such as still being highly dependent on UV activation or suffering from a high degree of hole–electron pair recombination that limits efficiency. As external factors, most studies have been conducted in controlled media that fail to capture the chemical complexity of real waters, where competing ions, organic matter, and fluctuating pH can significantly affect oxidation kinetics and catalyst reusability. Additionally, issues such as catalyst recovery, long-term stability, and scalability continue to hinder real-world implementation.

In general, although photocatalysis is positioned as a viable and environmentally friendly alternative for the remediation of As(iii), its transition to becoming an applicable water treatment technology will still require overcoming various challenges such as: the rational design of multifunctional materials with strong visible light activity, finding scalable and economical synthesis routes aligned with green chemistry principles, and a systematic evaluation of the catalysts under realistic conditions. Advances in computational modeling and interdisciplinary collaboration between materials science, environmental chemistry, and process engineering will be crucial to accelerate the transition of photocatalytic arsenic oxidation from laboratory research to real waters.

## Conflicts of interest

There are no conflicts of interest to declare.

## Data Availability

No new data or structures were created or analyzed in this study. All data analyzed came from previous publications and are appropriately cited in the manuscript.
